# Flower-Shaped CoS-Co_2_O_3_/G-C3N4 Nanocomposite for Two-Symmetric-Electrodes Supercapacitor of High Capacitance Efficiency Examined in Basic and Acidic Mediums

**DOI:** 10.3390/mi13122234

**Published:** 2022-12-16

**Authors:** Mohamed Rabia, Doaa Essam, Fatemah H. Alkallas, Mohamed Shaban, Samira Elaissi, Amira Ben Gouider Trabelsi

**Affiliations:** 1 Nanomaterials Science Research Laboratory, Chemistry Department, Faculty of Science, Beni-Suef University, Beni-Suef 62514, Egypt; 2 Nanophotonics and Applications Lab, Physics Department, Faculty of Science, Beni-Suef University, Beni-Suef 62514, Egypt; 3 Department of Physics, College of Science, Princess Nourah bint Abdulrahman University, P.O. Box 84428, Riyadh 11671, Saudi Arabia

**Keywords:** supercapacitor, cobalt sulfide, cobalt oxide, graphitic carbon nitride, composite

## Abstract

Graphitic carbon nitride (G-C3N4) was synthesized through the direct combustion of urea in the air. The CoS-Co_2_O_3_/G-C3N4 composite was synthesized via the hydrothermal method of G-C3N4 using cobalt salts. The morphological and chemical structures were determined through XRD, XPS, SEM, and TEM. XRD and XPS analyses confirmed the chemical structure, function groups, and elements percentage of the prepared nanocomposite. SEM measurements illustrated the formation of G-C3N4 sheets, as well as the flower shape of the CoS-Co_2_O_3_/G-C3N4 composite, evidenced through the formation of nano appendages over G-C3N4 sheets. TEM confirmed the 2D nanosheets of G-C3N4 with an average width and length of 80 nm and 170 nm, respectively. Two symmetric electrodes for the supercapacitor from the CoS-Co_2_O_3_/G-C3N4 composite. Electrochemical measurements were carried out to determine the charge/discharge, cyclic voltammetry, stability, and impedance of the prepared supercapacitor. The measurements were carried out under acid (0.5 M HCL) and basic (6.0 M NaOH) mediums. The charge and discharge lifetime values in the acid and base medium were 85 and 456 s, respectively. The cyclic voltammetry behavior was rectangular in a base medium for the pseudocapacitance feature. The supercapacitor had 100% stability retention up to 600 cycles; then, the stability decreased to 98.5% after 1000 cycles. The supercapacitor displayed a specific capacitance (C_S_) of 361 and 92 F/g, and an energy density equal to 28.7 and 30.2 W h kg^−1^ in the basic and acidic mediums, respectively. Our findings demonstrate the capabilities of supercapacitors to become an alternative solution to batteries, owing to their easy and low-cost manufacturing technique.

## 1. Introduction

Sustainable energy has become a promising field owing to cost-effective energy sources. Supercapacitors have been considered replacements for batteries. This is due to their simple charging, lasting a long time before being completely discharged [1,2]. Three types of supercapacitors, i.e., double-layer, hybrid, and pseudo-capacitors, have been developed so far. However, commercial supercapacitors are based on carbon materials (double-layer supercapacitors). Nevertheless, such capacitors have a large drawback related to the limited energy produced [3]. This has motivated researchers to develop advanced supercapacitors based on two composite carbon materials (hybrid supercapacitors). Indeed, the unique combination of composite materials ensures a better efficiency of supercapacitors that remains related to the redox reaction of the used material [4]. Particularly, the combination of carbon materials with redox materials in developing a supercapacitor ensures a significant enhancement of the capacitance through the charge storage inside the layers.

On the other hand, the electrochemistry of supercapacitors highly depends on the morphology of the used materials. For instance, using carbon layer materials decorated with additional metal oxides or sulfides increases the surface area that consequently originates extra active sites [5,6,7,8]. Consequently, this raises the charge storage inside the composite materials [3].

Graphitic carbon nitride (G-C3N4) is considered a promising material due to its simple preparation in a high-mass product, through the combustion of organic nitrogen materials such as urea and thiourea. G-C3N4 has great properties related to charge storage, represented by high surface area and charge chemical durability. This is assigned to the chemical structure of G-C3N4 of tris-triazine (C_6_N_7_) in the ternary amino group [9]. Santos et al. fabricated CoO and CuO decorated on G-C3N4; a specific capacitance (CS) of 124.75 and 84.28 at 0.5 A g were found, respectively [10]. Kuila et al. synthesized Gd decorated on G-C3N4 for supercapacitor applications; a capacitance equal to 2.59 mF cm^−2^ at 10 mV s^−1^ was obtained [11]. Furthermore, Rani et al. investigated the CoFe_2_O_4_/G-C3N4 composite, they demonstrated the impact of G-C3N4 on enhancing the electrical properties of the supercapacitor [12]. On the other hand, additional studies have been carried out on graphitic materials, such as mesoporous carbon nitride/graphene from 0.5 M H_2_SO_4_ as an electrolyte; a C_S_ of 240 F/g was produced [13]. Additionally, the Cs of G-C3N4/graphene electrode from 1 M KOH were studied and its value was equal to 265 F/g [14]. Similarly, a supercapacitor from 0.4 M LiClO_4_ demonstrated a C_S_ of 264 F/g [15]. Additionally, G-C3N4/carbon nanotube was used as the supercapacitor from a polyvinyl alcohol/H_2_SO_4_ electrolyte; the produced C_S_ value was 148 F/g [16]. G-C3N4/activated carbon from 1 M Na_2_SO_4_; the produced C_S_ value was 266 F/g [17].

Herein, a flower-shaped CoS-Co_2_O_3_/G-C3N4 nanocomposite for a two-symmetric-electrodes supercapacitor is prepared and tested in base and acid mediums. All the chemical analyses for confirming the chemical structure and morphology are carried out. The electrochemical properties are tested by measuring the charge/discharge, cyclic voltammetry, impedance, and stability of the prepared supercapacitor. The supercapacitor demonstrates a promising electrical property in the basic medium in comparison to the acid medium. The supercapacitor has a great performance in comparison to instances in the literature.

## 2. Experimental Section

### 2.1. Materials

Urea, thiourea, HCl, NaCl, and NaOH were purchased from Piochem Co., Cairo, Egypt. While cobalt acetate (Co(CH_3_COOH)_2_) and ammonia (NH_4_OH) were purchased from El Naser chemical Co., Cairo, Egypt. Nafion (5 wt.% dissolved in methanol) was obtained through Sigma–Aldrich, St. Louis, MO, USA.

### 2.2. Preparation of CoS-Co_2_O_3_/G-C3N4 Nanocomposite

First, 10 g of urea was well annealed in a tube furnace at 550 °C for 2 h under an N_2_ atmosphere. This led to the combustion and conversion of urea to G-C3N4.

For the preparation of the composite, (0.1 M) Co(CH_3_COOH)_2_, (0.2 M) thiourea, and (0.5 M) urea were dissolved in 40 mL H_2_O using an ultrasonic stirrer. Then, the pH solution was raised to 9 using an aqueous ammonia solution. Meanwhile, 0.05 g C3N4 was suspended in 30 mL H_2_O. Then, both solutions were added to each other inside the autoclave, in which the hydrothermal reaction was carried out at 160 °C for 12 h. After that, the produced powder was collected, washed, and dried at 60 °C for 5 h. Finally, the powder was annealed at 300 °C for 10 min. This process caused the formation of the CoS-Co_2_O_3_/G-C3N4 nanocomposite.

### 2.3. Synthesis and Electrochemical Testing of the Supercapacitor

Synthesis of the symmetric supercapacitor was carried out using the main prepared materials CoS-Co_2_O_3_/G-C3N4 nanocomposite. This material was used as a paste by mixing with (0.045 g) of nafion and 0.75 mL ethanol. Then, 0.003 g of this paste was loaded over an Au sheet (1 cm^2^). The supercapacitor was constructed by combining these two electrodes via a filter paper wetted with the electrolyte as a separator. Measurements were carried out under different electrolytes, such as HCl, NaOH, and NaCl, using an electrochemical workstation (CHI660E, USA) at 25 °C.

### 2.4. Characterization Process

The prepared materials were characterized using different analytical tools for confirming their chemical and morphological properties. X-ray diffraction (X’Pert Pro, Almelo Holland) and X-ray photoelectron spectroscopy were used to confirm the chemical structure of the prepared materials. The morphologies were determined using a scanning electron microscope, SEM, (ZEISS SUPRA 55 VP, Oberkochen, Germany) and a transmitted electron microscope (TEM), (JEOL JEM-2100).

## 3. Results and Discussion

**Figure 1:**
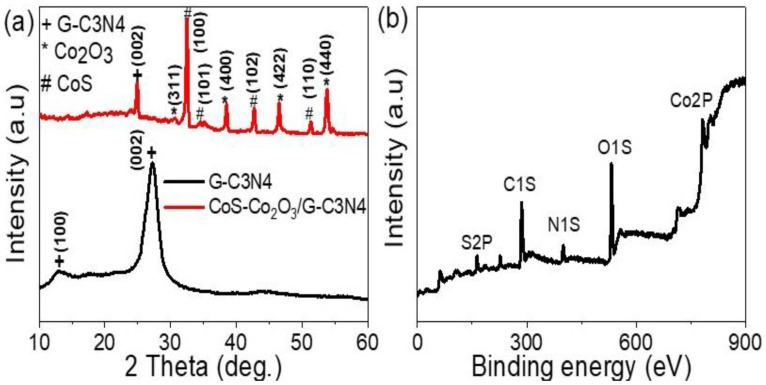
(**a**) XRD of G-C3N4 and CoS-Co_2_O_3_/G-C3N4 composite and (**b**) XPS of CoS-Co_2_O_3_/G-C3N4 composite.

The chemical and structural composition of the graphitic-like layer structures of G-C3N4 and CoS-Co_2_O_3_/G-C3N4 were confirmed using the XRD pattern (see Figure 1a). The G-C3N4 displayed two distinct diffraction peaks: a weak (100) diffraction peak at 13.03°, attributed to the in-planar structural packing motif with a separation of 0.679 nm, and a strong other peak located at 27.20° corresponding to the (002) peak of the interlayer d-spacing of 0.327 nm. The two peaks at 13.03° and 27.20° were matched with previous studies (JCPDS 87–1526 card) [18] and (JCPDS) 87–1526 for g-C3N4 [19]. The composite exhibited diffraction peaks corresponding to both g-C3N4 and CoS-Co_2_O_3_, reflecting the presence of two phases. The CoS nanomaterial had three peaks for the growth directions (101), (102), and (110), located at 32.45, 42.73, and 51.22°, respectively [20]. The Co_2_O_3_ nanomaterial had four peaks for the growth directions (311), (400), (422), and (440) for the positions 30.56, 38.51, 46.51, and 54.64°, respectively [21].

The chemical and structural composition was investigated via X-ray photoelectron spectroscopy (XPS) (see Figure 1b). Numerous elements were located in the sample, such as O, C, Co, N, and S (see Table 1). The O element was located at 532.5 eV and resulted from Co_2_O_3_ or oxygen inside the G-C3N4. Although the C1s spectrum was located at about 285.9 eV which can from the utilized pure G-C3N4, several bonds such as C-C, C-NH_2_, and N-C=N bonding at about 284, 286, and 288, respectively, were also distinguished. A small shift of the C-C XPS peak to higher binding energy was located, representing the chemical bonding between G-C3N4 and CoS-Co_2_O_3_. The N 1s strong peak centering at 399.7 eV was due to the nitrogen atom inside the G-C3N4. The S2p peak was located at 163.1 eV, which could be assigned to S-Co bonding. Furthermore, a Co2p spectrum peak was located at 782.25 eV, which could be assigned to the high binding between CoS-Co_2_O_3_ and G-C3N4.

**Figure 2:**
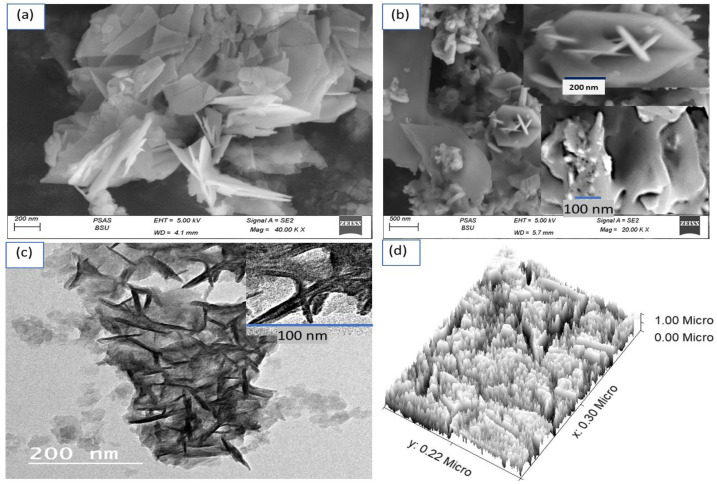
SEM of (**a**) G-C3N4 and (**b**) CoS-Co_2_O_3_/G-C3N4 composite. (**c**) TEM and (**d**) theoretical image of CoS-Co_2_O_3_/G-C3N4 composite.

The morphological and structural analysis of the samples were based on the correlation of SEM, TEM, and theoretical modeling images. Figure 2 represents SEM images of the (a) G-C3N4 and (b) CoS-Co_2_O_3_/G-C3N4 composites. The G-C3N4 has 2D nanosheet properties; the formed sheets enfold and crumple in some areas. The 2D nanosheets have an average width and length of 80 nm and 170 nm. It can be seen that both images reveal a thin sheet structure. Indeed, this was clearly distinguished through the TEM image (Figure 2c) of the G-C3N4, illustrating flat irregular shaped sheets with wrinkles. The dark area covering the sheets confirms CoS-Co_2_O_3_ incorporation in G-C3N4 sheets.

The theoretical modeling is shown in Figure 2d; the roughness and cross-section are clearly displayed. The formed CoS-Co_2_O_3_/G C3N4 composite is highly homogeneous roughness, in which the small particles coat the sheets represented by the G-C3N4.

### The Electrochemical Study of the Prepared Supercapacitor

**Figure 3:**
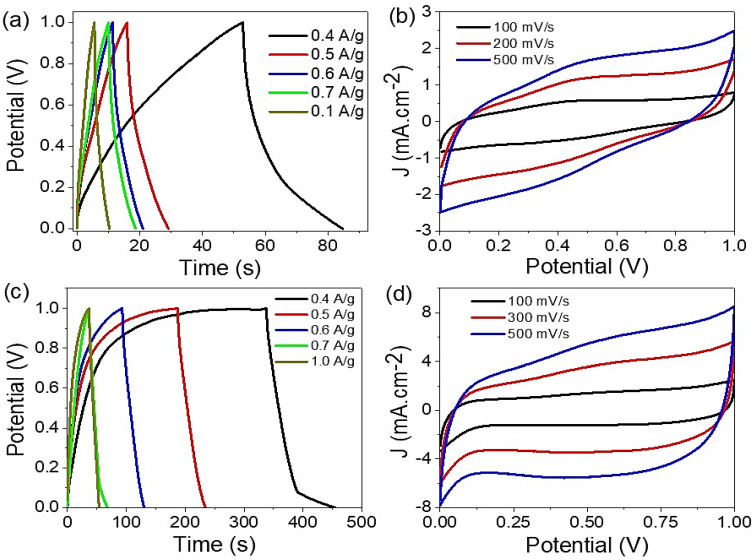
(**a**,**c**) Charge and discharge and (**b**,**d**) cyclic voltammetry curves for CoS-Co_2_O_3_/G-C3N4 nanocomposite supercapacitor under HCl and NaOH electrolytes, respectively.

The charge and discharge behavior of CoS-Co_2_O_3_/G-C3N4 composite nanomaterials were studied under different electrolytes; 6 M NaOH and 0.5 M HCL (see Figure 3a,b). Measurements were carried out in a potential window (0 to 1.0 V) at 25 °C, through a current density of 0.4 to 1.0 A/g. Moreover, the supercapacitor based on two symmetric structured electrodes was used, in which 0.0003 g paste was loaded over each electrode; then, a filter paper saturated with the selected electrolyte was used as a separator between these two electrodes. From the curve behavior, the capacitive properties affected the current density, which decreased with an increase in the inserted current density. Furthermore, the capacitive properties had a greater value in the basic medium in comparison with the acid medium. This is related to the effect of the HCl electrolyte on Co_2_O_3_ under the reaction of an acid with a base. The charge and discharge lifetime values in the acid and base mediums were 85 and 456 s, respectively. This confirmed the non-linear curves and change in behavior in the base medium [22,23,24]. The formation of the OH^-^ group caused increasing negative charge on the supercapacitor electrode that enhanced the pseudocapacitance properties of the prepared supercapacitor. On the other hand, the H^+^ ions caused the same effect, but the acid-base reaction under the presence of Co_2_O_3_ decreased the total capacitance.

The cyclic voltammetry behavior confirmed the charge and discharge behavior [25,26], where the produced current density values had a greater value in the base medium than in the acid medium (see Figure 3c,d). The rectangular shape appeared in the base medium, which confirmed the pseudo-capacitance feature [27,28], in which the surface area increased by increasing the scan rate from 100 to 500 mV.s^−1^. This confirmed the diffusion time for the electrolyte to diffuse inside the supercapacitor paste. The oxidation and reduction behavior increased on both sides of the cyclic voltammetry curve under the rectangular formed shape. The cyclic voltammetry behavior depends on the electrolyte used, in which the rectangular shape is confirmed from a base medium for the pseudo-capacitance feature.

**Figure 4:**
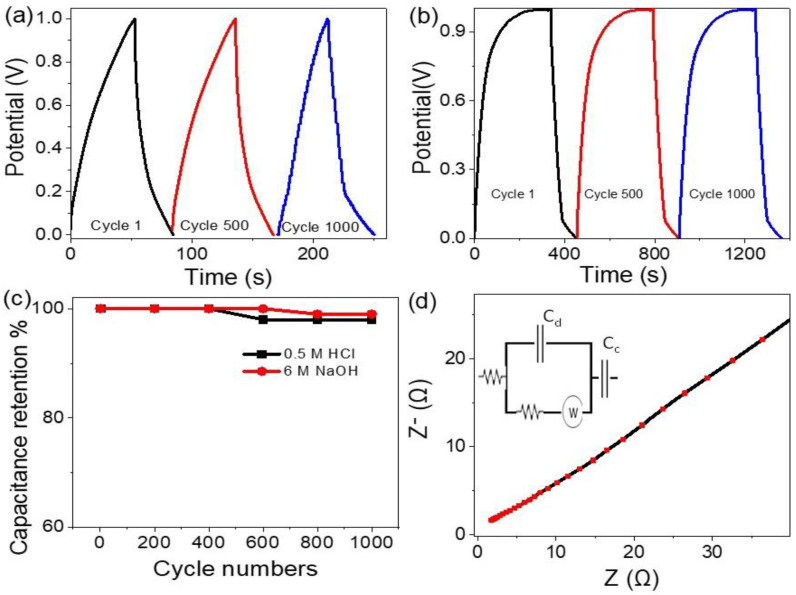
The cycle lifetime of the supercapacitor using the electrolytes (**a**) 0.5 M HCl and (**b**) M NaOH. (**c**) Capacitance retention using different electrolytes and (**d**) the impedance of the supercapacitor in basic medium 6 M NaOH.

The stability of the supercapacitor electrodes is determined through the charge and discharge behavior at a current density of 0.4 A/g for 1000 cycles using two different electrolytes, 0.5 M HCl and 6 M NaOH (see, Figure 4a,b). From these figures, the two supercapacitors in both the acid and base mediums have greater stability up to 1000 cycles, but the supercapacitor in the base medium obtains better behavior. Figure 4c gives the relation of cycle numbers and the capacitance retention in both acid and base mediums. From this figure, both supercapacitors have great stability up to 400 cycles, but then the supercapacitor in the acid medium faces a small stability loss, which may be due to the reaction of HCl with the Co_2_O_3_ inside the paste of the electrode. The supercapacitor in the base medium has 100% stability retention up to 600 cycles; then, the stability decreases to 98.5% up to 1000 cycles.

The relation between the real (Z) and imaginary (Z^−^) impedance (Nyquist plot) is shown in Figure 4d for the supercapacitor in 6 M NaOH. Moreover, the impedance circuit represents the electrochemical contact of the prepared supercapacitor (see insert Figure 4d), in which the series and charge transfer resistance represent the electrolyte and electrolyte/electrode resistances, respectively, while the Warburg impedance represents the resistance of the electrode material [29,30]. Moreover, the capacitance appears in the circuit that represents the contact. The supercapacitor displays a very small Z^−^ value, confirming the greater behavior of NaOH as an electrolyte for charge transfer (R_ct_) between the electrolyte and the electrode (see Figure 4d). R_CT_ characterizes the rate of redox reactions at the electrode/electrolyte interface, while C_dl_ occurs at the interfaces between solids and ionic solutions due to separations of ionic and electronic charges. The values of R_S_ and R_CT_ are 1.54 and 6.4 Ω, respectively, while the C_dl_ value is 18 µF.

**Figure 5:**
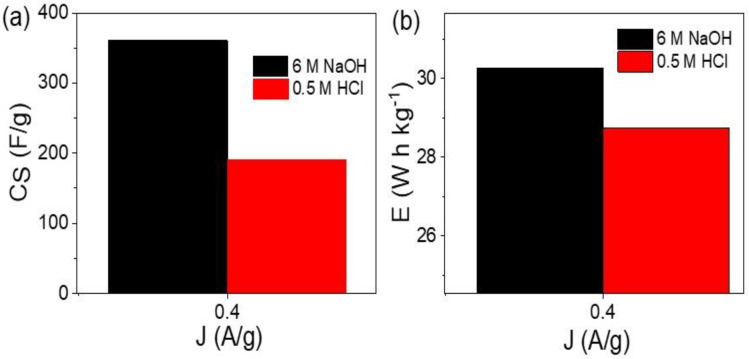
(**a**) The specific capacitance and (**b**) energy density for the prepared two symmetric electrodes supercapacitor in acid and base mediums.

For calculation of the performance of the prepared supercapacitor, the specific capacitance (C_S_) was determined in the acid and base mediums (see, Figure 5a). Such a calculation is based on Equation (1) [31,32], in which I, Δt, ΔV, and *m* represent current (A), discharge time (s), potential window (V), and the paste mass (g), respectively. The supercapacitor has C_S_ values of 361 and 92 F/g in the basic and acidic mediums, respectively. In addition, the energy density (*E*) represents the capacitance of the prepared supercapacitor. These E values were calculated for the supercapacitor from 6 M NaOH and 0.5 M HCl, as shown in Figure 5b and Equation (2). This *E* value depends on potential windows *V_max_* and *V_min_*, in which the produced E values in the acid and base mediums are 28.7 and 30.2 W h kg^−1^, respectively.

(1) $$C_{s}=4I.\Delta t/\;\Delta V.m$$

(2) $$E=0.5C_{\mathrm{sp}}.\;\left(V_{max\;}^{2}-V_{min\;\;}^{2}\right)$$

## 4. Conclusions

Flower-shaped CoS-Co_2_O_3_/G-C3N4 composite was prepared using the hydrothermal method for two-symmetric-electrodes supercapacitor application. Such a composite was based on G-C3N4, first prepared using the combustion process in ambient air. The morphological properties were confirmed through SEM and TEM analyses. The chemical structures were confirmed using XRD and XPS analyses, in which all the functional groups and elements were confirmed. The electrochemical studies were carried out by testing the prepared supercapacitor from the HCl and NaOH mediums. The supercapacitor parameters, charge/discharge, cyclic voltammetry, stability, and impedance of the prepared supercapacitor, were studied. The supercapacitor had a significant response in the basic medium in comparison to the acid medium. The supercapacitor had 100 and 98.5% stability retention up to 600 and 1000 cycles, respectively. The supercapacitor had C_S_ values of 361 and 92 F/g in the basic and acidic mediums, respectively. Moreover, the E values were 28.7 and 30.2 W h kg^−1^, respectively. This promising prepared supercapacitor device has great electrochemical properties that qualifies it for industrial applications.

## Figures and Tables

**Table 1:** The binding energy and percent of elements in the composite CoS-Co_2_O_3_/G-C3N4 were calculated from the XPS analysis.

Element	Peak (eV)	Atomic%
O1s	532.5	25.01
C1s	285.9	52.39
N1s	399.7	8.1
S2p	163.1	7.28
Co2p	782.2	7.22

## Data Availability

Not applicable.

## References

[1] Mai L.Q., Minhas-Khan A., Tian X., Hercule K.M., Zhao Y.L., Lin X., Xu X. (2013). Synergistic Interaction between Redox-Active Electrolyte and Binder-Free Functionalized Carbon for Ultrahigh Supercapacitor Performance. Nat. Commun..

[2] Krishnamoorthy K., Pazhamalai P., Mariappan V.K., Nardekar S.S., Sahoo S., Kim S.J. (2020). Probing the Energy Conversion Process in Piezoelectric-Driven Electrochemical Self-Charging Supercapacitor Power Cell Using Piezoelectrochemical Spectroscopy. Nat. Commun..

[3] Mai L.Q., Yang F., Zhao Y.L., Xu X., Xu L., Luo Y.Z. (2011). Hierarchical MnMoO4/CoMoO4 Heterostructured Nanowires with Enhanced Supercapacitor Performance. Nat. Commun..

[4] Chahal P., Madaswamy S.L., Lee S.C., Wabaidur S.M., Dhayalan V., Ponnusamy V.K., Dhanusuraman R. (2022). Novel Manganese Oxide Decorated Polyaniline/Graphitic Carbon Nitride Nanohybrid Material for Efficient Supercapacitor Application. Fuel.

[5] Rabia M., Mohamed H.S.H., Shaban M., Taha S. (2018). Preparation of Polyaniline/PbS Core-Shell Nano/Microcomposite and Its Application for Photocatalytic H_2_ Electrogeneration from H_2_O. Sci. Rep..

[6] Mohamed H.S.H., Rabia M., Zhou X.G., Qin X.S., Khabiri G., Shaban M., Younus H.A., Taha S., Hu Z.Y., Liu J. (2021). Phase-Junction Ag/TiO_2_ Nanocomposite as Photocathode for H2 Generation. J. Mater. Sci. Technol..

[7] Mohamed F., Rabia M., Shaban M. (2020). Synthesis and Characterization of Biogenic Iron Oxides of Different Nanomorphologies from Pomegranate Peels for Efficient Solar Hydrogen Production. J. Mater. Res. Technol..

[8] Shaban M., Rabia M., Fathallah W., El-Mawgoud N.A., Mahmoud A., Hussien H., Said O. (2017). Preparation and Characterization of Polyaniline and Ag/ Polyaniline Composite Nanoporous Particles and Their Antimicrobial Activities. J. Polym. Environ..

[9] Zheng Z.X., Wang M., Shi X.Z., Wang C.M. (2019). Palladium Nanoparticles/Graphitic Carbon Nitride Nanosheets-Carbon Nanotubes as a Catalytic Amplification Platform for the Selective Determination of 17α-Ethinylestradiol in Feedstuffs. Sci. Rep..

[10] Santos R.S., Suresh Babu R., Devendiran M., Haddad D.B., de Barros A.L.F. (2022). Facile Synthesis of Transition Metal (M = Cu, Co) Oxide Grafted Graphitic Carbon Nitride Nanosheets for High Performance Asymmetric Supercapacitors. Mater. Lett..

[11] Kumar Kuila S., Ghorai A., Midya A., Sekhar Tiwary C., Kumar Kundu T. (2022). Chemisorption of Gadolinium Ions on 2D-Graphitic Carbon Nitride Nanosheet for Enhanced Solid-State Supercapacitor Performance. Chem. Phys. Lett..

[12] Rani B., Nayak A.K., Sahu N.K. (2021). Electrochemical Supercapacitor Application of CoFe_2_O_4_ Nanoparticles Decorated over Graphitic Carbon Nitride. Diam. Relat. Mater..

[13] Nazari M., Rahmanifar M.S., Noori A., Li W., Zhang C., Mousavi M.F. (2021). The Ordered Mesoporous Carbon Nitride-Graphene Aerogel Nanocomposite for High-Performance Supercapacitors. J. Power Sources.

[14] Lin R., Li Z., Abou El Amaiem D.I., Zhang B., Brett D.J.L., He G., Parkin I.P. (2017). A General Method for Boosting the Supercapacitor Performance of Graphitic Carbon Nitride/Graphene Hybrids. J. Mater. Chem. A.

[15] Chen Q., Zhao Y., Huang X., Chen N., Qu L. (2015). Three-Dimensional Graphitic Carbon Nitride Functionalized Graphene-Based High-Performance Supercapacitors. J. Mater. Chem. A.

[16] Lu C., Chen X. (2020). Carbon Nanotubes/Graphitic Carbon Nitride Nanocomposites for All-Solid-State Supercapacitors. Sci. China Technol. Sci..

[17] Pilathottathil S., Kavil J., Shahin Thayyil M. (2022). Boosting Ion Dynamics by Developing Graphitic Carbon Nitride/Carbon Hybrid Electrode Materials for Ionogel Supercapacitor. Mater. Sci. Eng. B.

[18] Azizi-Toupkanloo H., Karimi-Nazarabad M., Shakeri M., Eftekhari M. (2019). Photocatalytic Mineralization of Hard-Degradable Morphine by Visible Light-Driven Ag@g-C3N4 Nanostructures. Environ. Sci. Pollut. Res..

[19] Kumar A., Kumar P., Joshi C., Manchanda M., Boukherroub R., Jain S.L. (2016). Nickel Decorated on Phosphorous-Doped Carbon Nitride as an Efficient Photocatalyst for Reduction of Nitrobenzenes. Nanomaterials.

[20] Kim J.H., Lee J.H., Kang Y.C. (2014). Electrochemical Properties of Cobalt Sulfide-Carbon Composite Powders Prepared by Simple Sulfidation Process of Spray-Dried Precursor Powders. Electrochim. Acta.

[21] Rajeevgandhi C., Sathiyamurthy K., Guganathan L., Savithiri S., Bharanidharan S., Mohan K. (2020). Experimental and Theoretical Investigations on the Spinel Structure of Co_2_O_3_ Nanoparticles Synthesized via Simple Co-Precipitation Method. J. Mater. Sci. Mater. Electron..

[22] Atta A., Abdelhamied M.M., Essam D., Shaban M., Alshammari A.H., Rabia M. (2021). Structural and Physical Properties of Polyaniline/Silver Oxide/Silver Nanocomposite Electrode for Supercapacitor Applications. Int. J. Energy Res..

[23] Gamal A., Shaban M., BinSabt M., Moussa M., Ahmed A.M., Rabia M., Hamdy H. (2022). Facile Fabrication of Polyaniline/Pbs Nanocomposite for High-Performance Supercapacitor Application. Nanomaterials.

[24] Different Types of Supercapacitors, 978-620-2-67505-5, 6202675055, 9786202675055 by Mohamed Mosaad Hamid, Eman Ali, Mohamed Rabia. https://www.morebooks.de/store/gb/book/different-types-of-supercapacitors/isbn/978-620-2-67505-5.

[25] Sayyah E.S.M., Shaban M., Rabia M. (2018). A sensor of m-cresol nanopolymer/Pt-electrode film for detection of lead ions by potentiometric methods. Adv. Polym. Technol..

[26] Sayyah S.M., Shaban M., Rabia M. (2018). Electropolymerization of m-toluidin on platinum electrode from aqueous acidic solution and character of the obtained polymer. Adv. Polym. Technol..

[27] Hao J., Huang Y., He C., Xu W., Yuan L., Shu D., Song X., Meng T. (2018). Bio-Templated Fabrication of Three-Dimensional Network Activated Carbons Derived from Mycelium Pellets for Supercapacitor Applications. Sci. Rep..

[28] Ramadan M., Abdellah A.M., Mohamed S.G., Allam N.K. (2018). 3D Interconnected Binder-Free Electrospun MnO@C Nanofibers for Supercapacitor Devices. Sci. Rep..

[29] Jones P.K., Stimming U., Lee A.A. (2022). Impedance-Based Forecasting of Lithium-Ion Battery Performance amid Uneven Usage. Nat. Commun..

[30] Qu D., Ji W., Qu H. (2022). Probing Process Kinetics in Batteries with Electrochemical Impedance Spectroscopy. Commun. Mater..

[31] Yu J., Fu N., Zhao J., Liu R., Li F., Du Y., Yang Z. (2019). High Specific Capacitance Electrode Material for Supercapacitors Based on Resin-Derived Nitrogen-Doped Porous Carbons. ACS Omega.

[32] Naveed ur Rehman M., Munawar T., Nadeem M.S., Mukhtar F., Maqbool A., Riaz M., Manzoor S., Ashiq M.N., Iqbal F. (2021). Facile Synthesis and Characterization of Conducting Polymer-Metal Oxide Based Core-Shell PANI-Pr_2_O–NiO–Co_3_O_4_ Nanocomposite: As Electrode Material for Supercapacitor. Ceram. Int..

